# Experimental Studies of PowerCore Filters and Pleated Filter Baffles

**DOI:** 10.3390/ma15207292

**Published:** 2022-10-18

**Authors:** Tadeusz Dziubak

**Affiliations:** Faculty of Mechanical Engineering, Military University of Technology, 2 Gen. Sylwestra Kaliskiego Street, 00-908 Warszaw, Poland; tadeusz.dziubak@wat.edu.pl

**Keywords:** PowerCore air filter, cylindrical air filter, separation efficiency, filtration performance, pressure drop, dust absorption coefficient

## Abstract

The material most commonly used to filter and clean the intake air of vehicle internal combustion engines is pleated filter paper, which in most cases is shaped in the form of a cylinder or panel. The production technology has a low cost and is not complicated. In addition to high separation efficiency and filtration performance, pleated filter media are required to have low initial pressure drop, which depends on the geometry of the bed. Much research has been conducted in this area. Dust accumulated in the filter bed causes an increase in pressure drop, which is the cause of deformation and sticking of pleats. The lack of stability of the pleats, the need to strengthen them, and the need to obtain small sizes while achieving high efficiency and accuracy of filtration of engine intake air was the reason for the development of a different design and a new technology for making filter cartridges called PowerCore. The distinctive feature of these filters is axial flow in one direction of the air stream, which avoids turbulence and thus minimizes pressure drop. This paper presents a comparative analysis of a standard PowerCore and PowerCore G2 filter bed and two cylindrical filters with a pleated filter bed made of cellulose and polyester. The conditions and methodology of experimental testing of filters with test dust are presented. During the tests, the characteristics of separation efficiency and filtration performance, as well as pressure drop as a function of the mass of dust retained on the filter of two PowerCore filters and two cylindrical filters were performed. Three specimens of test filters with the same filtration area were made from each sample of filter bed. The results showed that in each test of the filter bed, there is an initial filtration period characterized by low (96–98%) initial separation efficiency and the presence of large (*d_pmax_*) dust grains. As the dust loading of the bed increases, the separation efficiency and filtration performance obtain higher and higher values. The initial period of filtration ends when the conventional value (99.9%) of separation efficiency is reached. The duration of this period depends on the type of filter bed and for the PowerCore G2 filter ends for a dust loading of *k_m_* = 33.1 g/m^2^, and for the cellulose filter for *k_m_* = 117.3 g/m^2^. During the initial period, the air behind the PowerCore G2 filter contains grains with sizes in the range of *d_pmax_* = 9–16 µm. Behind the cellulose filter, dust grains are much larger, *d_pmax_* = 17–35 µm. The total operating time of the PowerCore G2 filter, limited by the achievement of the permittivity resistance Δ*p_wdop_* = 3 kPa, is twice that of the other filter compositions tested.

## 1. Introduction

Internal combustion engines, which are vehicles’ power sources, consume a significant amounts of atmospheric air when operating under rated conditions to ensure proper fuel combustion and achieve the required performance. Passenger car engines suck in 150–400 m^3^ of air per hour. For truck engines, this value is 900–2000 m^3^/h, and 3000–6000 m^3^/h for special vehicle engines (tanks, transporters).

Internal combustion engines of cars and special vehicles, including military vehicles, suck in significant masses of pollutants along with the air from the vehicle’s surroundings. Their main component is mineral dust, which is lifted from the dry ground by vehicles traveling at high speeds. The cause of dust lifting can also be blowing wind. Silica SiO_2_ and alumina Al_2_O_3_ are the main components of road dust, and their total share in dust reaches 95%. In addition, the dust contains: Fe_2_O_3_, MgO, CaO, K_2_O, Na_2_O and moisture [1,2].

Mineral dust from the substrate is polydisperse dust, that is, composed of particles of different sizes and different numbers. The indicator that characterizes the relative contribution of successive size groups of dust to its total mass is determined by its fractional composition, which depends on the type of substrate and atmospheric conditions. The fractional composition of dust changes with the height above the ground. This is determined by how long the dust grains remain in the air, which is directly related to the speed at which the grains fall. The speed at which grains fall depends on their mass, as well as their diameter and density. The speed of falling grains is determined by the force of gravity and the reciprocal relationship of aerodynamic drag force and gravity force. The falling velocity of grains increases significantly with an increase in their diameter. For example, SiO_2_ silica grains having diameters of 10 µm, 50 µm and 100 µm (density 2650 kg/m^3^) and fall at speeds of: 0.08 m/s, 0.19 m/s, 0.7 m/s [3,4], respectively.

Practically, polydisperse dust grains with diameters below the range of *d_p_* = 50–80 are found in the air around a moving vehicle. The mass share of these grains of the total dust mass is 80–100%. The presence of dust grains with diameters of more than 50 m is found in the air around special vehicles that operate on sandy training grounds, as well as around construction vehicles and machinery. Similar air pollution conditions can occur when a helicopter takes off or lands on a sandy dry area [5]. Air filter intakes for internal combustion engines usually draw in dust grains less than 100 µm in size.

The measure of dust concentration in the air is the mass of dust (in grams or milligrams) found in 1 m^3^ of atmospheric air. The concentration of dust in the air is a variable quantity and depends on many factors. In the case of a moving vehicle, the concentration of dust in its surroundings depends on the type of ground (sandy, loess), the speed of travel, the presence of other vehicles, weather conditions (rain, drought, wind direction), the type of running gear (wheeled, tracked) and the type of soils around the vehicle.

According to the author of the paper [6], the concentration of dust in the air takes on values ranging from 0.01 mg/m^3^ near rural buildings to about 20 g/m^3^ when a column of tracked vehicles travels over desert terrain. According to the author of the paper [7], dust concentrations in the air can take values in the range of 0.001–10 g/m^3^. Dust concentrations on highways take on small values, but in the wide range of 0.0004–0.1 g/m^3^, while when a column of vehicles moves over sandy terrain, dust concentrations take on much larger values of 0.03–8 g/m^3^ [8].

Airborne dust, due to its high hardness, which is formed by silica SiO_2_ and alumina Al_2_O_3_ dust grains, and whose mass content in the dust reaches 95%, is the main cause of accelerated abrasive wear of two frictionally mating machine components. In the case of internal combustion engines, these are the components of the T-P-C (piston—piston rings—cylinder walls) association. According to the hardness evaluated on the basis of the ten-point Mohs scale, silica has a hardness of 7, and alumina has a hardness of 9. The wear of engine components is mainly due to the abrasive action of particles with diameters in the range of 1–40 µm [9,10,11,12,13,14], with the highest intensity of wear caused by mineral dust grains in the diameter range of 1–20 µm [11]. It has been proven that the greatest wear of two mating machine surfaces is caused by dust particles *d_p_*, for which the condition is met at a given time: the diameter of *d_p_* is equal to the thickness of the oil film, *h_min_*. In the mating of a modern internal combustion engine, the thickness of the oil film, *h_min_*, between two surfaces takes values in the wide range of *h_min_* = 0.3–50 µm [15,16].

Excessive abrasive wear of the piston rings and cylinder liner of an internal combustion engine has a significant impact on the loss of tightness of this association, which is a direct cause of the loss of compressed medium and a decrease in compression pressure, and as a result there is a decrease in engine performance, a decrease in power and an increase in fuel and oil consumption [17,18].

In order to reduce friction losses and wear of the main components of an internal combustion engine, anti-wear coatings are applied to the surface of the piston rings and the cylinder surface [19]. Another factor in reducing friction losses in internal combustion engine associations is the continuity of the oil film, which is ensured by the proper shape of the sliding surfaces of the piston rings [20,21].

For a device that ensures the minimization of abrasive wear of engine components, an air filter is installed in the intake system, which ensures that air of the required purity, that is, free of dust grains above 2–5 µm, is supplied to the engine cylinders. In the intake systems of passenger car engines, which are operated with a low concentration of dust in the air, single-stage (baffle) filters are installed. Adequate air purity is then provided by a filter element made of pleated paper shaped into a rectangular panel. Engines of trucks, special vehicles (tanks, Infantry Fighting Vehicles (IFVs)), Armored Personnel Carriers (APCs) and work machines, which are most often used in conditions with high air dustiness, are equipped with two-stage filters operating in the “multicyclone-pore baffle” system [22,23].

The first stage of air filtration is a multi-cyclone, and the second is a cylindrical filter cartridge. Due to space limitations and the desire to increase the surface area of the filter material, filter cartridges are made of pleated filter paper.

A characteristic feature of baffle filters is the retention and deposition of dust particles on the filter cartridge, resulting in the flow of air into the engine that is free of dangerous contaminants. The impurities retained and accumulated in the filter baffle cause an increase in the packing density of the bed, resulting in a continuous increase in the value of the air filter pressure drop Δ*p_f_*. The intensity of the increase in filter pressure drop depends on the conditions under which the vehicle is operated, and mainly on the concentration of dust in the air and the duration of vehicle (engine) operation. An increase in air filter resistance leads to a decrease in engine power and increased fuel consumption, i.e., additional operating costs and an increase in exhaust emissions [24,25,26,27,28].

The operation of an air filter is a technical compromise between the ever-increasing pressure drop that is the cause of decreased power, increased fuel consumption and increased exhaust emissions of a vehicle engine and the high efficiency and accuracy of inlet air filtration, factors that determine the value of component wear, durability and reliability of the engine and the entire facility [29,30]. Hence, there are constant efforts of researchers to obtain an air filtration element with a low pressure drop, in order to reduce the adverse effects of the air filtration element on engine performance.

**Figure 1:**
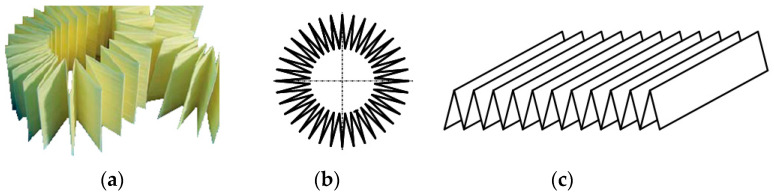
Shaping of filter paper: (**a**) paper after pleating, (**b**) shaping into a multi-armed star, (**c**) shaping into a panel.

Currently, the most common filter element used to filter the intake air of vehicle internal combustion engines is a filter element made of paper. Due to the limited space available to mount the filter in the space around the car engine, filter elements made of filter paper or nonwoven fabric are made in pleated form (Figure 1), from which the filter element can then be assembled in various ways.

In modern passenger cars, there is a problem with the proper location of the air filter in the engine environment—it is under the hood. Such a problem does not exist in the case of trucks, where the air filter and the entire intake system are mounted outside the vehicle cabin.

The continuous development of internal combustion engine design in the direction of increasing engine power and minimizing exhaust emissions has resulted in the fitting of new systems and equipment, for example: air conditioning systems with a compressor, turbochargers and exhaust gas recirculation systems with EGR valve. As a result of these measures, the space around the engine is reduced, with the result that there is not enough space to locate an air filter with adequate parameters.

Above all, there is the problem of using an adequate filter paper area, which results from the condition of permissible filtration speed, *υ_F_* = 0.08–0.12 m/s [31,32,33,34,35,36]. A smaller surface area of filter paper results while maintaining the same value of air flow with in an increase in the speed of air flow through the filter bed. As a result, there is an increase in air filter flow resistance, which is the direct cause of a decrease in engine fill and power, as well as an increase in exhaust emissions. Significantly exceeding the permissible speed can cause a decrease in filtration efficiency due to the phenomenon of particles bouncing off the bed surface and getting entrained again [37].

**Figure 2:**
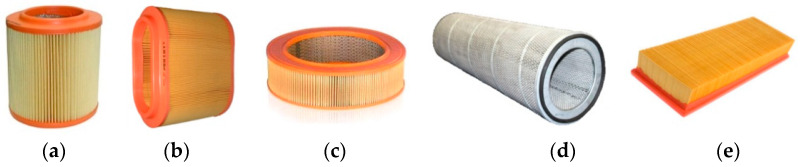
Types of paper air filter cartridges: (**a**) cylindrical with a circular base, (**b**) cylindrical with an oval base, (**c**) annular, (**d**) cylindrical conical, (**e**) panel.

Motor vehicle filter media can be constructed in the form of: (a,b) a cylinder with a circular or oval base, (c) a low-height ring, (d) a cone, (e) a rectangular panel (Figure 2).

**Figure 3:**
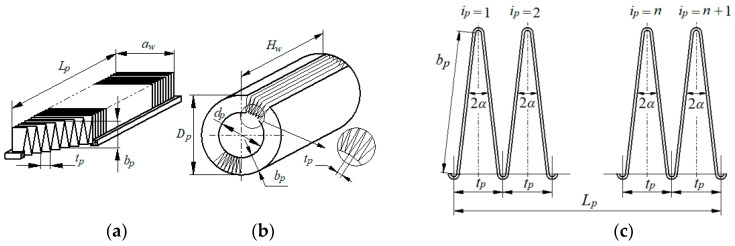
Geometry of the filter bed: (**a**) panel cartridge, (**b**) cylindrical cartridge, (**c**) pleat geometry. *L_p_*—the length of the cartridge, *a_w_*—the width of the pleat, *b_p_*—the height of the side of the pleat, *t_p_*—the pitch of the pleat (the gap between the pleats), *D_p_*—the outside diameter of the cartridge, *H_w_*—the height of the cartridge, *α*—the angle of the half pleat.

Filter cartridges, regardless of type, are made of pleated paper (cellulose) or a component of several materials with the appropriate requirements. It is necessary to obtain the largest filtration surface of the paper while meeting the condition of the maximum permissible filtration velocity, *v_Fmax_*, at a fixed minimum volume of the cartridge. This goal is achievable through appropriate selection of the main structural dimensions of the cartridge and the geometry of the pleats—Figure 3. The surface area of the filter paper is determined by the geometry of the pleats, including the number of pleats, their height and width, and the distance between the pleats.

For cylindrical cartridges, the filtration area is determined by the relationship:

(1) $$A_{c}=2H_{w}\cdot b_{p}\cdot i_{p}\left[m^{2}\right],$$

where *i_p_*—number of pleats determined from the relation:

(2) $$i_{p}=\frac{\pi \cdot d_{p}}{t_{p}}.$$

The design parameters of the cartridges are interrelated and must therefore be selected accordingly. In the case of cylindrical inserts, a restriction is adopted in the form of the height of the insert, which should not exceed *H_w_* = 400 mm. On the other hand, the height of the pleat *b_p_* should not be too high, because then the tendency of neighboring pleats to contact each other increases. For a given outer diameter, *D_p_*, of a cylindrical filter cartridge and a constant pleat spacing, *t_p_*, decreasing the inner diameter, *d_p_*, increases the pleat height, *b_p_*, and thus increases the filter paper surface area of the cartridge. However, decreasing the inner diameter, *d_p_*, causes a constant value, *t_p_*, of pleat (measured at the diameter *d_p_*—Figure 3) numbers to decrease, and, therefore, the surface area of the filter paper of the cartridge decreases. The maximum surface area of the filter paper of a cylindrical cartridge is obtained when the following condition is met:

(3) $$d_{p}=\frac{D_{p}}{2}.$$

**Figure 4:**
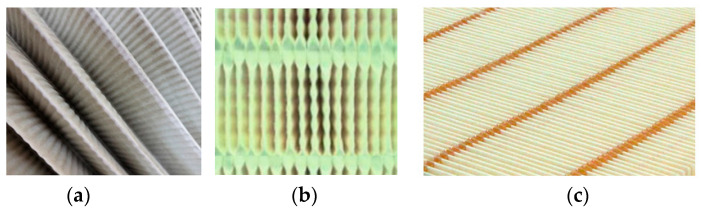
Ways to reinforce pleats: (**a**) pleat surface embossing, (**b**) special thickening of the back of the insert pleat, (**c**) glue trickles.

**Figure 5:**
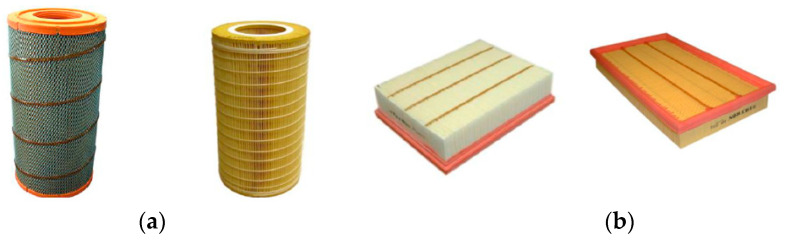
Example of reinforcement of pleats with glue streams: (**a**) cylindrical inserts, (**b**) panels.

During operation of the cartridge, the paper filter bed fills with dust, which is a significant impediment to the air stream flowing at high speed. There is a significant increase in pressure drop, and an outward sign of this phenomenon is the contacting and warping of pleats. To prevent this phenomenon, pleat reinforcements are used in the form of pleat surface embossing, special pleat ridge thickening (Figure 4a–c) and thin glue strips connecting the pleat ridges of the cartridge (Figure 4 and Figure 5).

The authors of the paper [38], while studying the properties of pleat geometry, found that the pressure drop and separation efficiency of the filter bed are affected not only by the geometry of the pleats (the shape of the pleat and its height and distribution density), but also by the geometric blocking effects of the pleats, as well as their stabilization technique, which is commonly used to maintain the pleat shape and the distance between them.

The lack of stability of pleats, the need to reinforce them as well as the need to meet the requirements of small size while maintaining or exceeding the efficiency and accuracy of filtration of engine intake air by filters manufactured to date, were the reasons for the development of a different filter design and new technology for the manufacture of filter cartridges, whose characteristic is the axial flow of the air stream. This avoids turbulence and allows the aerosol to flow directly to the filter outlet, thereby minimizing pressure drop.

**Figure 6:**
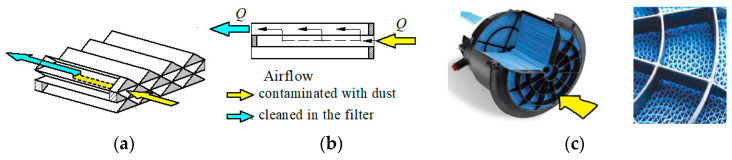
PowerCore filter cartridge: (**a**) filter cartridge principle, (**b**) direction of air flow, (**c**) front view of cartridge. The drawing was made by the author based on the data in [40].

An example of such a solution is the filter cartridge known as PowerCore (Figure 6). The PowerCore filter cartridge has a core design formed by alternating layers of smooth and pleated paper. The resulting channels are alternately blinded. The channel that has a free inlet is blocked on the outlet side and vice versa. Such a design forces air flow through the filter material into adjacent channels.

At the same airflow rate, filters made with PowerCore technology are dimensionally 2–3 times smaller than filters with pleated filter paper cartridges made with a conventional method, and are more efficient (*φ_f_* = 99.99%) than an average conventional filter, achieving an efficiency of *φ_f_* = 99.85% [39,40,41]. Higher filter capacity means longer filter life, which means less frequent filter changes and thus lower costs of operation.

**Figure 7:**
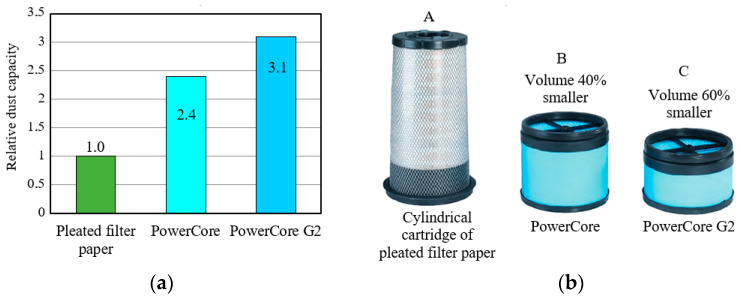
Comparison of PowerCore cartridges with respect to traditional (filter paper) filter media: (**a**) relative dust capacity, (**b**) occupied volume. The drawing was made by the author based on the data in [41].

The PowerCore filter cartridge allows for a 60% reduction in cartridge size and a nearly threefold increase in filter cartridge dust absorbency, compared to conventional solutions used to date, and with the same constant filter paper area and constant cartridge size (Figure 7).

The continued need for further downsizing and space restriction for intake air filters has led to innovations such as the PowerCore G2. The essence of this technology is the very precise shaping of the geometry of the filter core channels depending on the type of engine to which it is to be applied. Each geometric feature in the filter has been analyzed to reduce pressure losses and increase filter life (Figure 7). For a given filter life and performance, PowerCore G2 configurations can result in a size reduction of 30% compared to previous axial flow filters and 60% compared to cylindrical filters. Reducing the size of the filter element entails the possibility of reducing the size of the entire air filter.

**Figure 8:**
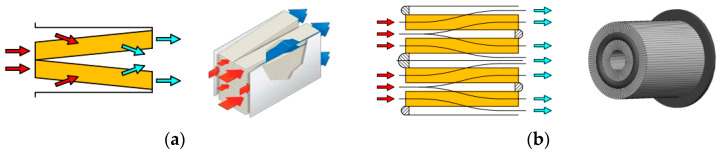
Principle of operation of Direct Flow filter cartridge: (**a**) panel, (**b**) cylindrical. The drawing was made by the author based on the data in [42].

Axial flow of the air stream, allowing flow directly to the filter outlet and allowing to minimize aerosol pressure drop, is provided by the Direct Flow filter cartridge manufactured by Cummins (Figure 8), in which the channels are formed by traditional pleats alternately sealed on its shorter sides and shaped in the form of panels [42]. The filter cartridge can be constructed in the form of a trapezoid of two panel cartridges set at a slight angle (Figure 8a) or in the form of two cylinders set coaxially (Figure 8b). Such a design forces air to flow through the pleats from the front and along the longer sides and outflow air on the opposite side.

**Figure 9:**
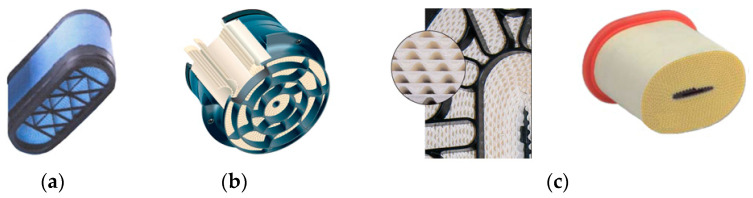
Filter cartridges made with modern technologies: (**a**) traditional PowerCore (Donaldson) [41], (**b**) Channel Flow (Baldwin) [43], (**c**) CompacPlus (Mann + Hummel) [44]. The drawing was made by the author based on the data in [41,43,44].

PowerCore cartridge technology was used by Baldwin in the Channel Flow filter (Figure 9b).

The PowerCore filter element reduces the space occupied by the filter by up to 50% compared to conventional cylindrical filters. The same technology used by Mann and Hummel in the PicoFlex filter has resulted in the CompacPlus filter element having 50% more filter medium surface area than a conventional air filter. Despite the obvious advantages that PowerCore filters show over conventional cylindrical cartridges, however, they are not yet widely used for engine intake air filtration due to their considerable (several times) cost. Therefore, work is still being carried out to optimize the geometric parameters of pleated filter beds, mainly in the direction of minimizing pressure drop, which will result in increased engine performance. At the same time, pleated paper filter cartridges should be designed in such a way that, given the cartridge design dimensions and the required filter paper surface area, they have maximum separation efficiency, minimum pressure drop, maximum durability and occupy the smallest possible volume.

There are no comparative analyses of the filter characteristics of PowerCore filters and cylindrical pleated cartridges in the available literature. Such characteristics can only be obtained during experimental tests on a special test dust bench. Experimental testing of inlet air filter beds of internal combustion engines is labour-intensive and expensive, but it is the most reliable research method. The aim of this study was a theoretical and experimental comparative evaluation of the filtration properties of PowerCore and pleated deposits.

## 2. Research and Optimization of Pleated Deposits in the Literature

An air filter element the most commonly used in the engine intake system is made of pleated filter paper. Reducing the pressure drop through the air filter element, and thus improving engine performance, was the subject of macroscopic optimization and geometric parameter studies of the fibrous pleated filter element. According to the conducted studies, a pleated filter bed with fixed geometric parameters (pleat height and width, pleat spacing) is characterized by the optimal number of pleats at which the minimum pressure drop was recorded [45,46,47,48,49,50,51,52,53,54,55,56,57,58,59,60,61,62].

For example, numerical studies of clean filters by Fotovati et al. [45] showed that regardless of the orientation of the fibers in the filter bed, there is an optimal number of pleats for which the filter flow resistance reaches a minimum. At the same time, triangular pleats cause less pressure drop than rectangular pleats.

Of great importance for the increase in flow resistance is the dust loading of the filter bed. As the number of pleats increases, the intensity of the increase in pressure drop decreases. A larger number of pleats in the filter bed, i.e., a smaller spacing between them, causes an increase in the flow velocity inside the pleat channels. This results in greater inhomogeneity of dust deposition on the surface of the pleats. This effect is less significant when the pleats of the filter bed are triangular in shape.

Similarly, Mahesh [46], performing an experimental and computational analysis of fluid flow through the pleated medium of an internal combustion engine air filter, showed that a filter bed made of triangular pleats achieves higher filtration efficiency and lower flow resistance than a filter bed having rectangular pleats.

In contrast, the authors of [47] determined the pressure drop of clean pleated filter beds. During the tests, the geometry of pleats was changed as follows: the distance between pleats in the range of 1–3.5 mm, pleat heights for values of 27, 32, 40, 48 mm and filtration velocity from 0.01 to 0.1 m/s. The study showed that for a fixed pleat height and a constant air flow, there is such a width between pleats for which the pressure drop reaches the smallest value. Optimization of the geometry of pleated deposits of motor vehicle filters was performed using the developed dimensionless model.

Park et al. [48] conducted research and optimization toward minimizing the flow resistance of several samples of pleated filter beds for varying the angle between pleats, different pleat lengths and the number of pleats. A dimensionless pleat factor was defined, which is the quotient of pleat height and pleat pitch (distance between the tops of successive pleats). The highest filtration efficiency was obtained when this coefficient reached a value of 1.48. After exceeding this value, a systematic increase in filter flow resistance was registered.

On the other hand, Saleh and Tafreshi [49], performing numerical studies toward minimizing pressure drop and obtaining maximum efficiency of various filter beds, showed that air filters with rectangular pleats can, at high dust loads, provide better performance than their triangular counterparts. These conclusions apply to filter performance in both depth and surface filtration regimes with particles of 1; 5 and 10 µm in diameter and in the filtration velocity range of 0.5–5 m/s.

Jin-rui et al. [50] built a three-dimensional pleated air filter model based on a real meltblown fiber. Various parameters of the filter bed were taken into account, namely: the angle contained between pleats, the thickness of the filter bed, the diameter of the fibers and their number. Simulation of the filtration characteristics of the pleated bed showed that, at the same inlet velocity, the pressure drop increases nonlinearly as the number of particles deposited on the fibers increases.

Maddineni et al. [37] numerically investigated the phenomenon of dust particle penetration through a pleated filter bed made in the form of a panel with the following parameters: pleat wall height of 26 mm, pleat pitch of 4.5 mm and angle between pleat walls of 2.5 degrees. After several tests with ISO 12103 A2 fine dust, an increase in particle penetration was found due to particle bouncing and retention at speeds above 0.5 m/s.

Li et al. [51] experimentally investigated the pressure drop of pleated air filters depending on the geometry of the pleats and the dust retained on them. They studied six filter test beds with different pleat ratios while keeping the side length of the pleats unchanged. The study showed that the effective filtration area was affected by the pleat ratio, so it should be kept below 1.59.

Chen et al. [52] conducted simultaneous optimization toward minimizing pressure and obtaining maximum separation efficiency of the pleated bed by adopting, as an evaluation criterion, the filtration quality factor, which combines the two parameters by the following relationship, whereby a higher *q*-factor indicates more efficient filtration of the bed.

(4) $$q=\frac{-\ln\left(1-\varphi_{c}\right)}{\Delta p}[1/\mathrm{kPa}]$$

where *φ_c_*—separation efficiency of the cyclone, and ∆*p*—pressure drop for nominal air flow [kPa].

The presented research showed that an increase in the number of pleats of the filter bed first causes an increase in the value of the quality factor *q*, and then its decrease. It was shown that this is influenced by two parameters: the number of pleats of the filter bed and their height. The study showed that the filtration efficiency of pleated beds is affected not only by dust parameters (grain size, density), but also by the number of pleats in the bed. The highest filtration quality coefficient was achieved by a bed with pleats of 2.65 per 10 mm with an influx of 122 nm diameter particles.

In contrast, Théron et al. [53] numerically and experimentally evaluated the effect of pleat geometry on the properties of fiber filters in the deep filtration phase of submicron aerosols. Wiegmann et al. [54] simulated a pleated air filter with different pleat shapes and filter materials and evaluated their effects on pressure drop.

In [26], a pleated filter bed with various parameters was studied experimentally and numerically, with the goal of optimization directed at minimizing pressure drop and improving the performance of a compression-ignition engine. The effects of several bed parameters were investigated, including the height and shape of the pleat and pleat spacing, the thickness of the filter bed as well as the effects of filtration speed and bed dust loading on engine speed, engine torque and fuel consumption. Three different pleat shapes were used during the tests. The lowest flow resistance was obtained for a filter with sinuous pleats, and the highest for a bed with a flat pleat shape. Reduced pleat spacing additionally resulted in higher (by about 18%) flow resistance, while increased pleat height or pleat thickness led to a reduction in pressure drop of about 43% and 10%, respectively. The dust-laden filter bed was the cause of a greater pressure drop and thus higher fuel consumption at the same engine torque and speed values. The operation of the engine equipped with the filter with optimized bed was characterized by the lowest (218 °C) exhaust gas temperature. The exhaust gas temperature of the engine operating with the standard filter reached a much higher value of 233 °C. The highest exhaust gas temperature of 250 °C was recorded for the engine operating with a dirty air filter.

Rebai et al. [55] found that the optimal number of pleats derived from pressure drop tests of clean-state filters was much lower than that based on the mass of retained dust in the filter. Fotovati et al. [56] studied the effect of pleated bed geometry on the mechanism of dust deposition in the pleats, as well as on the associated pressure drop and separation efficiency. They conducted numerical studies by keeping the pleat height constant.

Feng and Long [57] conducted an optimization of a pleated bed filtration process involving dust using macro-scale CFD. They proved that an optimal pleated density in a clean filter bed can result in higher pressure drop during the filtration process involving dust. This is associated with higher energy losses.

For example, in [58], numerical prediction of pressure drop in an air filter with a pleated filter element was carried out. It was shown that pleat geometry and inlet velocity are the basic parameters for filter element optimization. It was found that the optimal pleat pitch, at which the minimum pressure drop was obtained, was significantly dependent on the pleat height.

In another study [59], the authors optimized the pleat geometry using the filtration quality factor *q*, taking into account the separation efficiency and pressure drop of the filter. In this regard, they studied eight pleated filter beds (4, 6, 8, 10, 15, 21, 25, 30 pleats) with pleat height and width, respectively: 29 mm and 105 mm. It was shown that at the same air flow rate for filter beds with the number of pleats above 21, the flow resistance increased with a higher number of pleats. This phenomenon should be explained by an increased filtration rate resulting from a decrease in the effective surface area of the filter bed. The highest value of the filtration quality factor, *q* = 5.8, was obtained for a filter bed with a pleat height of 29 mm.

In the paper [60], the results of a numerical study of cellulose filter beds with different shapes (flat, W-shape and sinuous shape) were presented. It was shown that the filter element with a sinusoidal shape has a lower pressure drop, a more uniform distribution of the flow field and a smaller space size, which is the optimal solution. Experimental studies have shown that an engine equipped with an optimized filter element has lower specific fuel consumption compared to a pleated filter element, the lowest exhaust gas temperature at the same engine torque and a longer service life. The dust retention capacity of the optimized filter element is 16.2 g higher than the pleated filter element.

**Figure 10:**
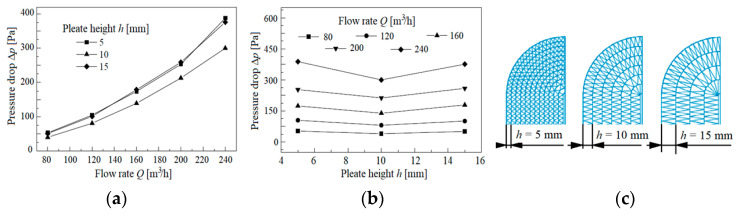
Change in pressure drop as a function of: (**a**) flow rate at different pleat heights, (**b**) pleat heights at different flow rates, (**c**) filter bed structure. Figure made by the author based on data from [61].

In [61], optimization of the air filter housing and filter bed with a honeycomb structure described by different pleat heights—*h* = 5, 10 and 15 mm—was carried out (Figure 10). Four variants of the air filter housing differing in shape of the inlet and outlet (circular or elliptical) with the same filter bed with a pleat height of *h* = 5 mm were studied. It was found that the filter housing with an elliptical inlet and circular outlet has the lowest pressure drop. Regardless of how the air flow rate changed in the range of 80–240 m^3^/h, the lowest pressure drop was obtained for a filter bed with a pleat height of *h* = 10 mm and optimized housing. This is due to the fact that an increase in pleat height (with the same pleat spacing) forces the angle between the sides of the pleats to decrease, which makes the air flow in the pleat channels more turbulent and increases the frictional resistance of the air inside the pleat channels.

## 3. Own Research

### 3.1. Aim, Scope and Subject Matter of the Study

The purpose of the study was to determine the following filtration properties: separation efficiency and filtration performance, as well as pressure drop of PowerCore research filters, and to compare them with cylindrical filters made of pleated material (cellulose, polyester) with known and different parameters, which are listed in Table 1. With similar thickness of the filter material, the cellulose material has six times higher permeability and much lower grammage than the polyester filter material, which may affect the filtration efficiency. During the experimental study, the following filtration characteristics were determined as a function of the dust absorption coefficient—*k_m_*.

- Filtration performance *d_pmax_* = *f*(*k_m_*),
- Separation efficiency *φ_w_* = *f*(*k_m_*),
- Pressure drop Δ*p_w_* = *f*(*k_m_*).

The dust absorption coefficient ratio, *k_m_*, was defined as the total mass of dust, *m_z_*, retained and evenly distributed over 1 m^2^ of the active area *A_w_* of the filter material and was expressed by the relation:

(5) $$k_{m}=\frac{m_{z}}{A_{w}}\;[g/m^{2}].$$

**Table 1:** Parameters of the structure of the tested filter materials given by the manufacturer.

Parameters	Filter Paper Identification	
	C	P
Type of filter material	Cellulose	Polyester
Air permeability *q_p_* [m^3^/m^2^/h] at 200 Pa	3015	540
Air permeability *q_p_* [dm^3^/m^2^/s]	838	150
Grammage *g_m_* [g/m^2^]	121	180
Thickness *g_z_* [µm]	610	550

Before and after the dust tests, the flow (aerodynamic) characteristics, Δ*p_w_* = *f*(*Q_w_*) of the filter media were performed, where *Q_w_* is the air flow rate through the filter cartridge.

**Figure 11:**
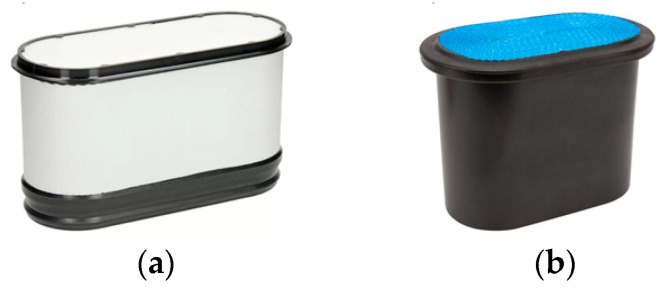
Original PowerCore filter cartridges used to make the test cartridges: (**a**) PowerCore—standard, (**b**) PowerCore G2 (author’s photos).

**Figure 12:**
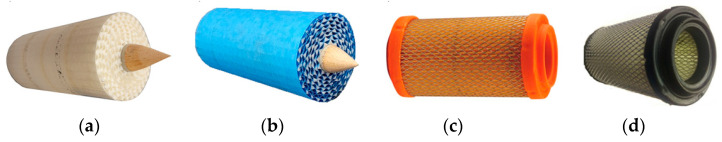
Test filters prepared for testing: (**a**) PowerCore filter—PC, (**b**) PowerCore G2 filter—G2, (**c**) filter with cellulose bed—C, (**d**) filter with polyester bed—P (photos taken by the author).

The subjects of the study were cylindrical filters and PowerCore filters. Cylindrical filters were made on the basis of a mass-produced passenger car filter cartridge with filter area of *A_w_* = 0.153 m^2^. Two different filter materials were used: cellulose and polyester. Deposits from two original commercially available PowerCore standard and PowerCore G2 filter cartridges were used to make PowerCore research filters (Figure 11). The cartridges were shaped to have the same filtration area (*A_w_* = 0.153 m^2^) as the cylindrical cartridges. For ease of analysis of the test results, the following test filters were conventionally designated: the PowerCore filters—PC and G2, respectively. The filter made of cellulose was designated—C, and the filter made of polyester was designated—P (Figure 12).

### 3.2. Methodology and Conditions of Experimental Research

**Figure 13:**
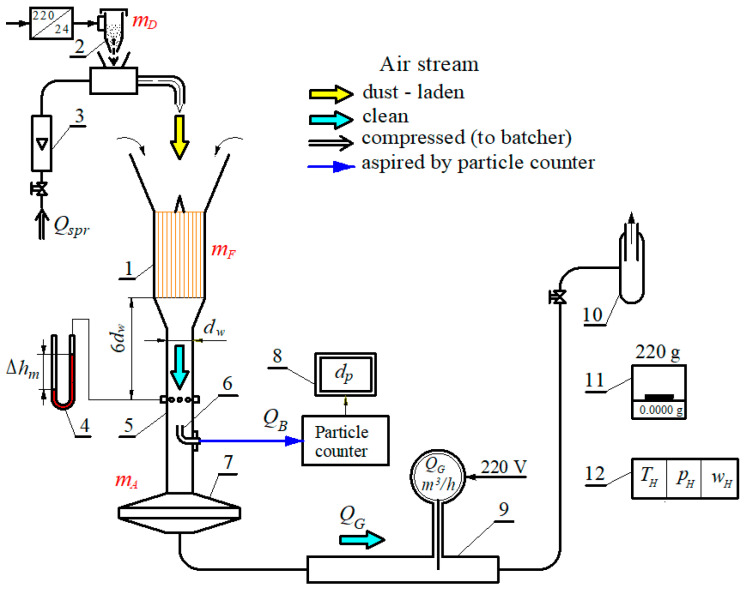
Diagram of the test stand for testing air filters of automotive engines: 1—filter cartridge, 2—dust dispenser, 3—rotameter, 4—U-tube type liquid pressure gauge for measuring pressure drop across the filter [mm H_2_O], 5—measuring tube, 6—measuring probe, 7—absolute filter, 8—measuring computer, 9—air flow meter, 10—suction fan, 11—analytical balance, 12—instrument for recording ambient air temperature and humidity and ambient pressure.

The tests were carried out on a test stand (Figure 13), which was equipped with a Pamas—2132 particle counter with an HCB-LD-2A-2000-1 sensor. The counter records the number and size of dust grains in the air stream, *Q_w_*, behind the tested filter cartridge in the range 0.7–100 µm in *i* = 32 measurement intervals, limited by diameters (*d_pimin_* − *d_pimax_*). A SensyMaster FMT430 thermal mass flow meter with a measuring range of 10–150 m^3^/h was used to measure the air flow, *Q_w_*. Test dust of the appropriate concentration is dispensed into the dust chamber by a vibrating dust dispenser. It is possible to obtain a dust concentration of up to 4 g/m^3^ in the intake air during testing. The stand prepared in this way allows for the testing of air filters with the original panel and cylindrical cartridges for engines of passenger cars or other vehicles with a maximum air demand of *Q_max_* = 80–150 m^3^/h. These are usually engines with a displacement of *V_ss_* = 0.6–1.0 dm^3^. The presented stand also allows testing of air filters with panel and cylindrical cartridges for engines of motor vehicles or other machines with air demand up to *Q_max_* = 350 m^3^/h. This requires the use of a flow meter with a measuring range of 100–500 m^3^/h, which is on the equipment of the laboratory. The required air flow is provided by under-pressure fans.

**Figure 14:**
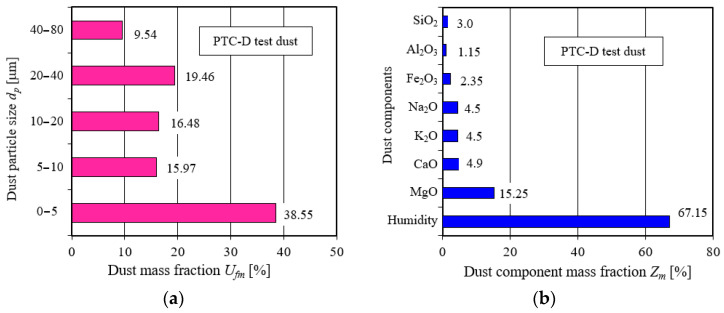
PTC-D test dust: (**a**) mass proportion of individual fractions in dust, (**b**) mass proportion of components in dust. Drawings made by the author based on data from [62].

The *Q_B_* test air flow to the particle counter sensor is drawn (aspirated) with the tip of a measuring probe placed centrally in the center of the measuring tube at a suitable distance behind the filter under test. The measuring line is terminated with a special measuring filter (absolute filter), on which the dust not trapped by the filter under test is deposited. The absolute filter is used to determine the separation efficiency according to relation 7. At the same time, the filter protects the air flow meter from dust entering the sensor. The test dust used was PTC-D, and the chemical and fractional composition, which is shown in Figure 14, is a substitute for the AC fine test dust in Poland. The mass proportion of the grains of the test dust used, with a size of 0–5 µm, is almost 40% of the total dust mass. These are dust grains that, due to their very small diameters, present great difficulty for retention by filtration mechanisms in porous filter materials. From the chemical composition of the dust shown in Figure 14, it can be seen that silica SiO_2_ is the primary component of the dust, and its mass proportion in the dust is more than 67%. In addition, it is a mineral with a high hardness (7 on the Mohs scale). Thus, it is the primary component of dust that is responsible for the accelerated wear of machine components.

The flow characteristics Δ*p_w_* = *f*(*Q_w_*) of the test filters were determined for eight measurement points in the airflow range *Q_w_* = *Q_wmin_* − *Q_wmax_*. The maximum flux value *Q_wmax_* was determined for an assumed maximum filtration velocity *υ_Fw_* = 0.1 m/s.

This value of filtration speed occurs in filters for the airflow produced by the motor at the maximum power speed *n_N_*.

For the bed filtration velocity adopted for the study (*υ_Fw_* = 0.1 m/s), the maximum value of the test flux, calculated using the following relationship, is *Q_wmax_* = 56 m^3^/h.

(6) $$Q_{wmax}=A_{w}\;\cdot v_{Fw\;}\cdot 3600\;[m^{3}/h].$$

The separation efficiency characteristics *φ_w_* = *f*(*k_m_*) of the test filters were determined by the mass method for a constant filtration speed, *υ_Fw_* = 0.1 or 0.06 m/s in successive *j* measurement cycles with a specified duration, *τ_p_*, or the time of uniform dust dosing to the filter. After each measurement cycle, the mass of dust was determined with an analytical balance: retained on the tested filter *m_F_*, on the absolute filter *m_A_* and the mass of dust dosed *m_D_* onto the filter. The dust concentration in the inlet air to the test filter, *s* = 0.5 g/m^3^, was used. The duration (time of uniform dust dosing) was set to *τ_p_* = 2 min in the initial (first I) period and *τ_p_* = 4 min in the main (second II) period of operation of the test filters.

During the measurement cycle (60 s before the planned end of the measurement), the procedure for measuring the number and size of dust grains in the air behind the filter was started in the particle counter.

After each measurement cycle, j was determined by:

1. The pressure drop Δ*p_fj_* of the filter, as the static pressure drop upstream and downstream of the filter based on the measured (after dust dosing) height Δ*h_mj_* on a water manometer (U-tube type) using the relation:
  (7) $$\Delta p_{f}=\frac{{\Delta h}_{m}\cdot g\cdot \rho_{w}}{1000}[\mathrm{Pa}]$$
  where Δ*h_m_*—the height of the column of the manometric liquid [mm], *g*—acceleration of gravity [m/s^2^] and *ρ*—density of the manometric liquid (water) [kg/m^3^].
2. Filter separation efficiency, which was defined as the quotient of the mass of dust, *m_Fj_*, retained by the tested filter and the mass of dust, *m_Dj_*, introduced with the inlet air into the filter during the subsequent *j* measurement cycle based on the relationship:
  (8) $$\varphi_{j}=\frac{m_{Fj}}{m_{Dj}}=\frac{m_{Fj}}{m_{Fj}+m_{Aj}}100\%.$$
3. Dust absorption coefficient, *k_mj_*, of the tested filter material:
  (9) $$k_{mj}=\frac{\sum_{j=1}^{n}m_{Fj}}{A_{w}}\;[g/\;m^{2}].$$
4. The number *N_zi_* of dust grains in the air stream downstream of the filter (passed through the filter material) in measurement intervals bounded by diameters (*d_pimin_* − *d_pimax_*).
5. Filtration performance as the largest dust grain size *d_pj_* = *d_pmax_* in the air stream downstream of the filter.
6. The percentage of individual dust grain fractions in the air behind the filter for a given test cycle:
  (10) $$U_{pi}=\frac{N_{pi}}{N_{p}}=\frac{N_{pi}}{\sum_{i=1}^{32}N_{pi}}100\%,$$
  where $N_{p}=\overset{32}{\underset{i=1}{\sum }}N_{pi}$—the total number of dust grains (average of five counts) that were passed through the test filter (covering all measurement ranges) during the test cycle.

The research was conducted in test cycles. Each cycle included five registrations (counts) of dust grain diameters in the range of 0.7–100 μm, in which 32 measurement ranges limited by fixed particle diameters (*d_pmin_ − d_pmax_*) were distinguished, consecutively.

## 4. Analysis of Research Results

**Figure 15:**
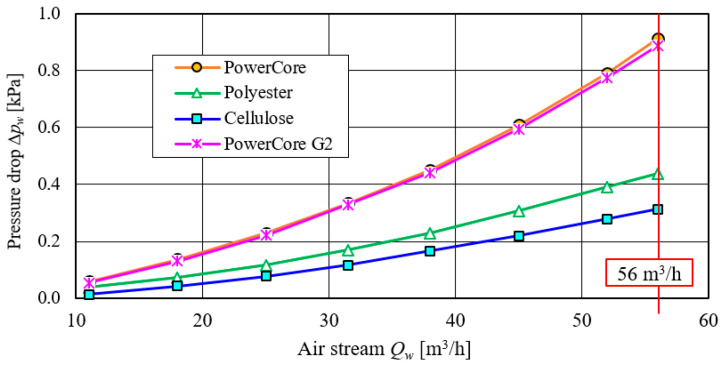
Flow characteristics, Δ*p_w_* = *f*(*Q_w_*), of filter media before testing with test dust.

The results of the flow characteristics, Δ*p_w_* = *f*(*Q_w_*), of the test filters are shown in Figure 15. As the air flow rate increases, there is a parabolic increase in the pressure drop of the test filters, which is consistent with the literature information.

The smallest value of pressure drop over the entire range of test airflow, *Q_w_*, was recorded for a filter with cellulose filter material. For *Q_wmax_* = 56 m^3^/h, the flow resistance has a value of ∆*p_w_* = 312 Pa (Figure 15). A slightly higher value is achieved by a filter made of polyester. The highest values of flow resistance over the entire range of the test air flow rate, *Q_w_*, were registered for PowerCore filters. For *Q_wmax_* = 56 m^3^/h, the pressure drop of both PowerCore filters is at ∆*p_w_* = 0.89–0.91 kPa (Figure 15).

**Figure 16:**
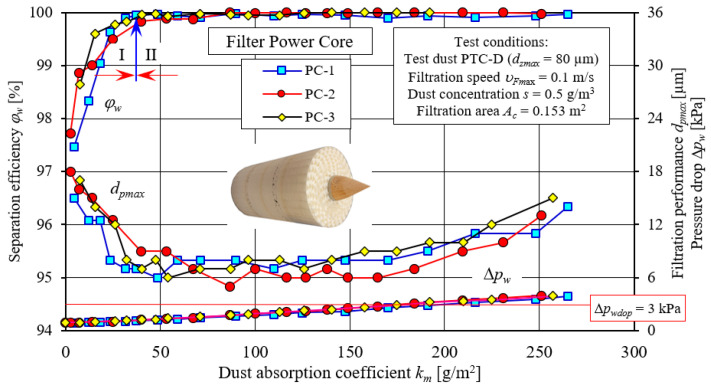
Characteristics of separation efficiency (*φ_w_*) and filtration performance (*d_pmax_*) and pressure drop (Δ*p_w_*) depending on the dust absorption coefficient (*k_m_*) of three test specimens of PowerCore-PC filter cartridges.

The results of testing the filtration characteristics of the separation efficiency *φ_w_* = *f*(*k_m_*) and filtration performance *d_pmax_* = *f*(*k_m_*), as well as the pressure drop Δ*p_w_* = *f*(*k_m_*) of the three specimens of PowerCore research filters (PC) are shown in Figure 16. The characteristics of successive specimens of PC filters differ slightly as to their course and values, and their course is similar and consistent with the information reported by many authors who have studied baffled filter media [63,64].

With the increase in the mass of dust retained in the filter layer (increase in the km coefficient), the separation efficiency and filtration performance, as well as the pressure drop of the PC filters, take on increasing values, except that the initial values and the intensity of change are slightly different for each filter. The initial separation efficiency of the tested filters is in the range *φ_w_* = 97.5–98.6%. At this time, there are dust grains of *d_pmax_* = 15–18 µm in the air behind the filter. With the influx of a mass of dust on the filter and the start of the filtration process, there is a sharp increase in separation efficiency and a decrease in the size of dust grains, *d_pmax_*. It was assumed during the study that as soon as the separation efficiency *φ_w_* = 99.9% is reached, the initial (I) filtration period ends. The following filtration period is called the primary (II) period—Figure 16. The value *φ_w_* = 99.9% is the minimum required separation efficiency of fibrous materials of motor vehicle engine filters.

In the case of the PC filters tested, the conventional separation efficiency, *φ_w_* = 99.9%, was obtained for an absorption coefficient in the range of *k_m_* = 40–50 g/m^2^, and the air behind the filter contains dust grains with a size of *d_pmax_* = 7–9 µm. The change in the filtration characteristics of separation efficiency *φ_w_* = *f*(*k_m_*) and filtration performance *d_pmax_* = *f*(*k_m_*) and pressure drop Δ*p_w_* = *f*(*k_m_*) is due to the filtration mechanisms in the filter bed, resulting in the retention of dust particles on the fiber surface of the porous structure and on previously deposited particles. They thus form slowly growing complex dendritic structures (agglomerates), which fill the free spaces between the fibers. This leads to a decrease in the free clearance between fibers, and thus an increase in air velocity, and, consequently, an increase in pressure drop.

Stage two (II) of the filtration process in the tested PowerCore porous baffle is characterized by a course of separation efficiency (*φ_w_* = 99.9%) and filtration performance (*d_pmax_* = 7–9 µm) stabilized at a certain level, and a slow increase in pressure drop. As soon as the assumed pressure drop Δ*p_w_* = 3 kPa was reached, the filters achieved a dust absorption coefficient *k_m_* of 200 g/m^2^. This value is comparable to the results of tests on other filter materials [34,65].

**Figure 17:**
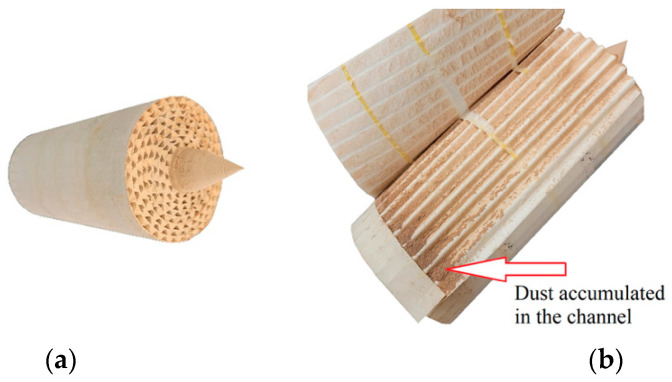
PowerCore filter after testing: (**a**) inlet view, (**b**) view of exposed channels.

The characteristic of PowerCore filters is that dust is retained not only in the space of the filter bed, but inside the channels plugged at the outlet—Figure 17.

**Figure 18:**
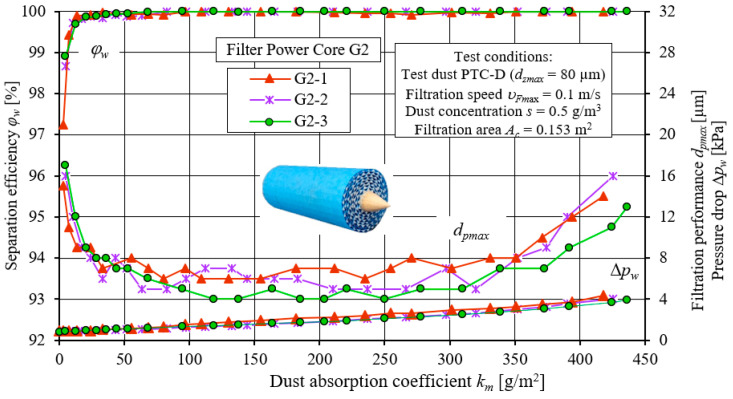
Characteristics of separation efficiency (*φ_w_*) and filtration performance (*d_pmax_*) and pressure drop (Δ*p_w_*) depending on the dust absorption coefficient (*k_m_*) of three test specimens of PowerCore—G2 filter cartridges.

**Figure 19:**
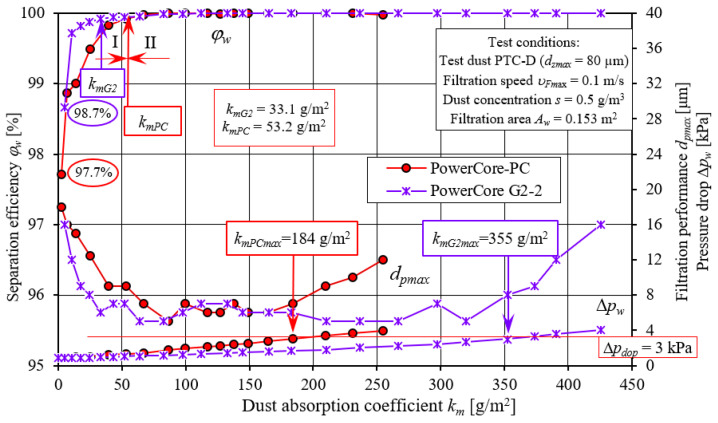
Characteristics of efficiency (*φ_w_*) and filtration performance (*d_pmax_*) and pressure drop (Δ*p_w_*) depending on the dust absorption coefficient (*k_m_*) of PowerCore—PC and PowerCore—G2 research filter cartridges.

Figure 18 shows the results of testing the characteristics of separation efficiency *φ_w_* = *f*(*k_m_*), and filtration performance, *d_pmax_* = *f*(*k_m_*), as well as pressure drop, Δ*p_w_* = *f*(*k_m_*), of three test copies of PowerCore G2 filters. The presented characteristics of successive copies of the filters differ slightly in their course and values. Their course is similar to the characteristics of the PowerCore filter cartridges (PC), with the PowerCore (G2) filters achieving significantly higher dust absorption. The exact differences in the characteristics of the two PowerCore research filters (PC and G2) are shown in Figure 19. The characteristics of the separation efficiency *φ_w_* and filtration performance, *d_pmax_*, as well as the pressure drop, Δ*p_w_*, of one of the three copies of the PC and G2 filters were used for analysis.

The initial separation efficiency of the PowerCore cartridges tested takes on varying values, with *φ_wPC_* = 97.7% and *φ_wG2_* = 98.7%, respectively (Figure 19). As the mass of dust retained in the filter layer increases (the increase in the km coefficient), the separation efficiency of the tested cartridges takes on increasing values, with the increase in the efficiency of the G2 cartridge being more intense. Therefore, the established value of separation efficiency (*φ_w_* = 99.9%—the end of period I) of the G2 cartridge is already reached after the dust absorption coefficient, *k_mG2_* = 33.1 g/m^2^. For the PC cartridge, the first stage takes slightly longer and ends at *k_mPC_* = 53.2 g/m^2^.

The operation of both cartridges is also differentiated by the duration of the second (II) period of the filtration process, which is characterized by high (*φ_w_* = 99.9 separation) efficiency and high filtration accuracy. During this time, the size of maximum dust grains takes on values at *d_pmax_* = 5–7 µm.

With the amount of dust mass dispensed onto the filter cartridge, the pressure drop increases its value, with the intensity of the increase being much lower for the G2 cartridge. The established value of Δ*p_dop_* = 3 kPa is reached by the PC cartridge at the dust absorption coefficient *k_mPC_* = 184 g/m^2^. For the G2 cartridge, this value is almost twice as high, *k_mPC_* = 325 g/m^2^. This should be explained by the properties of the PowerCore G2 filter bed. From the data presented in [41], it can be seen that the PowerCore G2 filter bed makes it possible to reduce the size of the filter cartridge and increase the dust absorption of filter cartridges, compared to conventional solutions used to date and with the same constant surface area of the filter paper, a certain service life and high separation efficiency.

**Figure 20:**
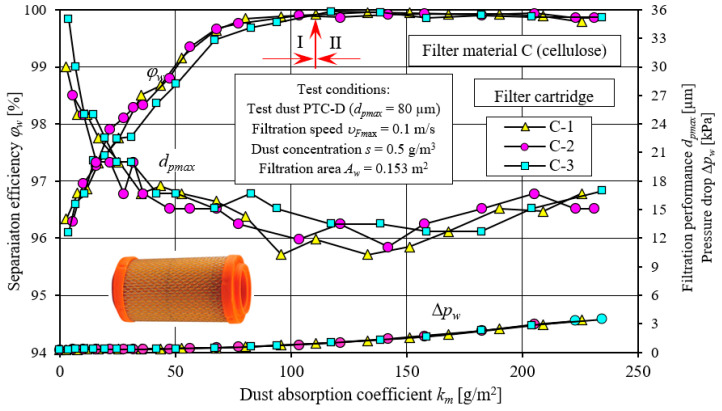
Characteristics of separation efficiency (*φ_w_*) and filtration performance (*d_pmax_*) and pressure drop (Δ*p_w_*) depending on the dust absorption coefficient (*k_m_*) of three copies of cylindrical filter cartridges made of cellulose—C.

**Figure 21:**
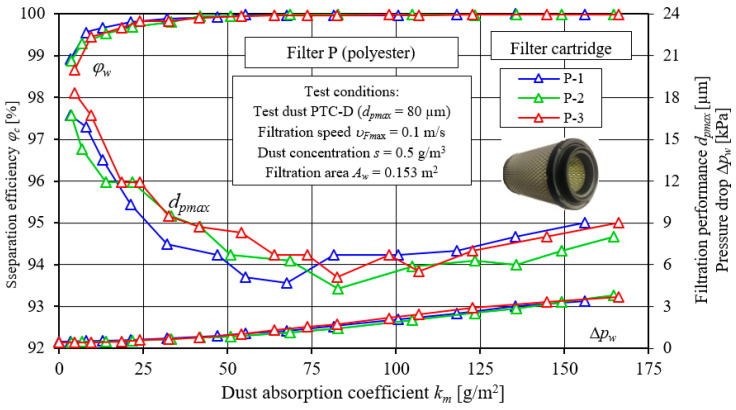
Characteristics of separation efficiency (*φ_w_*) and filtration performance (*d_pmax_*) and pressure drop (Δ*p_w_*) depending on the dust absorption coefficient (*k_m_*) of three copies of cylindrical filter cartridges made of polyester—P.

The next stage of testing involved the performance of filtration characteristics of two cylindrical filters: with a cellulose filter bed—C and with a polyester filter bed—P. The results of testing the characteristics of separation efficiency, *φ_w_* = *f*(*k_m_*), and filtration performance, *d_pmax_* = *f*(*k_m_*), as well as pressure drop, Δ*p_w_* = *f*(*k_m_*), of the three test specimens of filter cartridges C and P are shown in Figure 20 and Figure 21.

**Figure 22:**
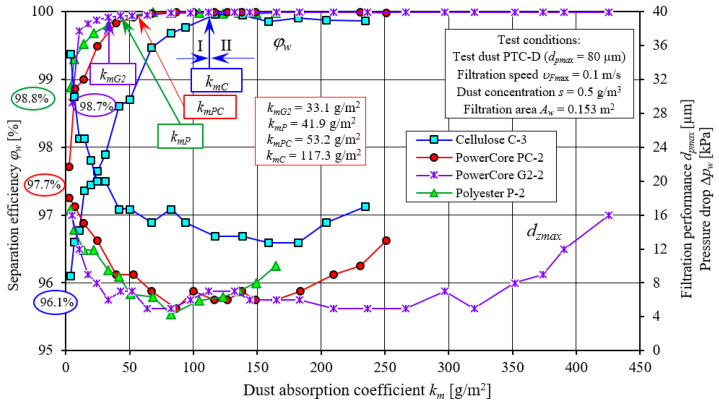
Characteristics of separation efficiency (*φ_w_*) and filtration performance (*d_pmax_*) depending on the dust absorption coefficient (*k_m_*) of the tested PC, G2, C and P filters.

**Figure 23:**
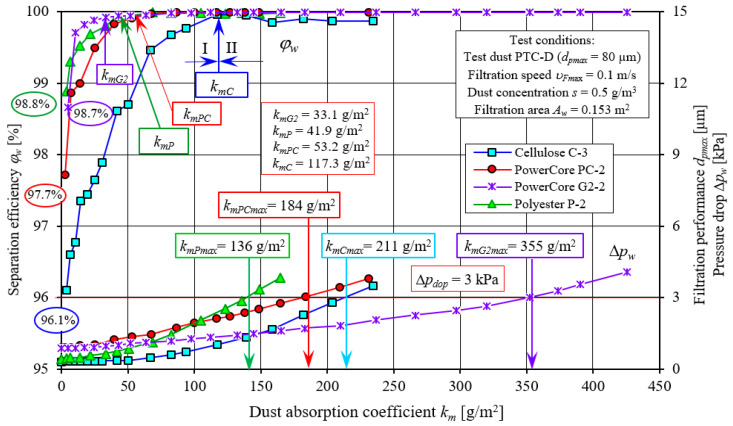
Characteristics of separation efficiency (*φ_w_*) and pressure drop depending on the dust absorption coefficient (*k_m_*) of the tested PC, G2, C and P filter cartridges.

The presented characteristics of successive copies of C and P filters differ slightly in their course and values. Their course is similar to the test characteristics of the PowerCore filter cartridges. Figure 22 and Figure 23 show a comparative analysis of the characteristics: separation efficiency *φ_w_* = *f*(*k_m_*), filtration performance *d_pmax_* = *f*(*k_m_*) and pressure drop Δ*p_w_* = *f*(*k_m_*) of PowerCore filter cartridges (PC, G2) and cylindrical filter cartridges (C and P). The characteristics of separation efficiency *φ_w_* and filtration performance *d_pmax_* and pressure drop Δ*p_w_* of one of the three copies of PC, G2, C and P cartridges were used for analysis.

The characteristics shown have a similar course but differ markedly in value. The differences recorded between the PC, G2, C and P filters are due to the type of filter material used and the technique of shaping it. Particularly large differences exist between the characteristics obtained for cartridge C (cellulose) and those of the other PC, G2 and P cartridges.

The initial separation efficiency of each of the tested cartridges takes on varying values. For the filter made of filter material C (cellulose), the lowest value (*φ_w0C_* = 96.1%) of initial separation efficiency was obtained. For the other filters, the values of initial efficiency are respectively higher: *φ_w0P_* = 97.7%, *φ_w0PC_* = 98.8% and *φ_w0G2_* = 98.7% (Figure 22). The intensity of the increase in separation efficiency until the target separation efficiency of *φ_w_* = 99.9% is significantly lower for filter C (cellulose) than for the other filters tested. Therefore, the time after which filter C reaches the assumed separation efficiency (initial period I) is much longer. Filter C reaches an efficiency of *φ_w_* = 99.9% after the dust absorption coefficient *k_mC_* = 117.3 g/m^2^. The initial period (I) of the C filter accounts for more than 50% of its total operating time. For the G2, P and PC filter made of other filter materials, the first stage (I), i.e., the time to achieve a separation efficiency of *φ_w_* = 99.9%, takes much less time. The dust absorption coefficient then reaches correspondingly smaller values: *k_mG2_* = 33.1 g/m^2^, *k_mP_* = 41.9 g/m^2^ and *k_mPC_* = 53.2 g/m^2^. For G2, P and PC filters, the initial period represents 9%, 31% and 29% of its total operating time, respectively.

With the start of the filtration process (period I), the sizes of the maximum dust grains behind the tested filters (in the purified air) take on varying values. The largest sizes of dust grains (*d_pmax_* = 35 µm) were registered behind the C (cellulose) cartridge, and the smallest, *d_pmax_* = 16–18 µm, behind the G2 and P filters, which is closely related to the initial separation efficiency of these cartridges. With the influx of dust grains on the filter bed and their deposition on the surface of the fibers, the structure of the bed changes, as a result of which its packing density increases. As a result, there is an increase in separation efficiency and accuracy and an increase in pressure drop. There are smaller dust grains in the air behind the filter. After the end of the 1st filtration period, the grain sizes are at *d_pmax_* = 12–15 µm (filter C) and *d_pmax_* = 7–9 µm (filters G2, PC and P)—Figure 22.

The initial stage (I) of filter operation, which is characterized by low separation efficiency and accuracy, has a definite impact on the life of the engine. Low separation efficiency and filtration performance in the initial stage of filter operation practically occurs after the replacement of a contaminated filter element with a new one in the engine intake system. The engine intake air then contains dust grains of considerable size, which cause accelerated wear of engine components, mainly the piston, piston rings and cylinder liner. According to the authors of works [9,10,11,12,13], all dust grains with a size of more than 1 µm are the cause of wear of the two specific cooperating machine parts. Filters for the intake air of internal combustion engines are required to have a filtration performance of more than 5 µm. It follows that the filter cartridge should not be replaced too often but only when this is due to its filtration properties. For motor vehicle users, the criterion for replacing the air filter is the achievement of the established permissible resistance. For passenger car engines, this value is Δ*p_dop_* = 3 kPa.

From the data in Figure 23, it can be seen that the tested filters P, PC and C achieve the established value of Δ*p_dop_* = 3 kPa in a similar time (at the dust absorption coefficient): *k_mP_* = 136 g/m^2^, *k_mPC_* = 184 g/m^2^, *k_mC_* = 203 g/m^2^, respectively. The established value of Δ*p_dop_* = 3 kPa is achieved by the G2 filter obtaining twice the value of the dust absorption coefficient *k_mPC_* = 355 g/m^2^. After the filter achieves the value of Δ*p_dop_* = 3 kPa, its operation is still possible, as the filters still achieve high (*φ_w_* = 99.9%) separation efficiency, but after some time, dust grains of increasingly larger sizes, reaching *d_pmax_* = 17 µm, begin to appear in the air behind the filter—Figure 23.

This phenomenon, characteristic of the process of filtration of aerosols in fibrous partitions, should be explained by the processes which take place in the fibrous partition during the influx of air with dust. As dust grains continuously flow in and are retained and deposited on the surface of individual fibers, agglomerates are formed, which, growing to a considerable size, fill the free spaces (pores) between the fibers. Dust grains that flow onto the filter bed are retained on the surface of individual fibers as a result of several basic filtration mechanisms: inertial, direct hooking, gravitational, diffusive, electrostatic and sieve. The growing agglomerates reduce the free field of air flow, which, at the same value of the air flow through the filter bed, causes an increase in velocity between the fibers. As a result, there is an increase in pressure drop that is a function of velocity squared. As the mass of dust accumulated in the filter bed increases, the pressure drop increases, as shown in Figure 23. Filter papers have a limited dust absorption capacity (the thickness of typical filter media papers is in the range of *g_p_* = 0.4–0.8 mm), and when this is exhausted, the dust settles on the surface of the bed impeding air flow, which manifests itself in an additional increase in flow resistance. At the same time, the high resistance of the air filter (significant vacuum generated behind the filter) and the high velocity of air flow in the spaces between the fibers, can cause local detachment of dust grains from the agglomerates formed and their migration towards the outlet. In the process of filtration in fiber baffles, this phenomenon is called “secondary emission.” In the case of the filter materials studied, the onset of this process occurred when the pressure drop value reached about ∆*p_f_* = 3 kPa.

Continued operation of the air filter is possible as the filtration process in the bed progresses, which is characterized by a higher intensity of increase in flow resistance. Increasingly, there will be local “secondary emission”, which will result in an increased number of large dust grains in the air downstream of the filter and a slight decrease in filtration efficiency. In extreme cases, the high flow resistance of the filter can cause mechanical destruction (breakage, tear apart, rip) of the filter material (paper) from which the filter element was made. In engine practice, this is a very unfavorable phenomenon, since dust grains will flow into the engine cylinders in greater numbers and sizes, which will result in accelerated wear of P-PR-C components. Hence, there is a need to replace the filter element when a certain flow resistance is reached, which is called the permissible resistance, Δ*p_dop_*. This is an operational parameter of the air filter, which is easily measured on a running engine. For each engine with certain parameters (e.g., engine cylinder displacement and its RPM) and the air filter working with it, the permissible resistance, Δ*p_dop_*, has a different value. For the vehicle user, it is the value of the permissible resistance that is important, which determines the need to perform filter maintenance—replacing the filter element with a new one. Determining such values of flow resistance was one of the objectives of this work. In the case of the tested air filters, their operation should be terminated when the value of Δ*p_dop_* = 3 kPa is reached.

**Figure 24:**
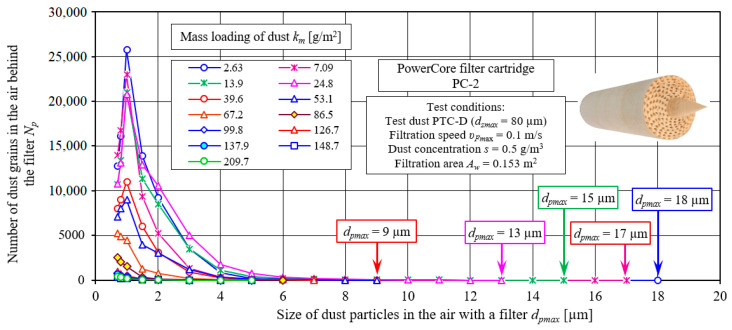
The number of dust grains in the measurement intervals recorded in the air behind the PC-2 filter for successive values of the dust absorption coefficient *k_m_*.

In the filtered air (behind the filter cartridge), there are dust grains of different sizes (*d_p_*) and in different numbers (*N_p_*). As the size, *d_p_*, of dust grains in the air behind the filter increases, their number, *N_p_*, first increases sharply, and the size *d_p_* = 1.0 µm reaches a maximum *N_pmax_*, and then decreases parabolically (Figure 24). With the amount of dust mass retained on the filter (the increase in the dust absorption coefficient *k_m_*), the number of dust grains in the air behind the filter decreases. Thus, the maximum *N_pmax_* takes on smaller values. Such a distribution of dust grains behind the filter occurs only until the filter reaches a dust loading of *k_m_* = 53.1 g/m^2^, that is, during the initial period (I) of the PC filter operation. In the second stage (II) of the filtration process, as the size (*d_p_*) of dust grains in the air behind the filter increases, the number of *N_p_* is smaller and smaller, which indicates the increasing separation efficiency of the tested material. In the last measurement interval, there is usually one dust grain with the largest size, *d_p_* = *d_pmax_*, which is the criterion for filtration accuracy. Example values of grain size *d_pmax_* are shown in Figure 24. As the mass of dust retained on the filter increases (the dust absorption coefficient *k_m_* increases), the size of dust grains, *d_pmax_*, in the air behind the filter takes on smaller and smaller values, as shown in Figure 22.

**Figure 25:**
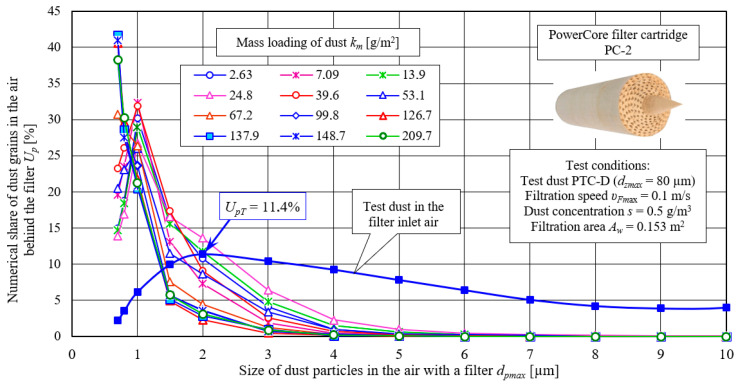
The proportion of *U_p_* dust grains in the air upstream and downstream of the PowerCore filter (PC-2) after reaching successive dust absorption coefficients *k_mPC2_*.

The proportion of *U_p_* dust grains in the air downstream of the PowerCore filter (PC-2) after the successive dust absorption coefficients *k_mPC2_* are shown in Figure 25. For comparison, the granulometric composition of the test dust, which is fed with the inlet air to the filters under test, is shown. The inlet air to the filter contains dust with grain sizes up to 80 µm. The largest share (*U_pT_* = 11.4%) of the test dust is made up of grains with a size of 2 µm. Below and above this value, the share of grains steadily decreases. The granulometric composition of dust grains in the air behind the filter has a completely different course. The filter bed primarily retains dust grains above *d_p_* = 5 µm, so in the air behind the filter the largest share is accounted for by grains with sizes below this value.

The maximum share of *U_p_* grains in the air behind the filter shifted towards smaller values and occurs at *d_p_* = 1.0 µm (Figure 25). After the first measurement (*k_m_* = 2.63 g/m^2^), the maximum share of dust grains *d_p_* = 1.0 µm has a value of *U_p1_* = 30.2%, In subsequent measurements, as the dust absorption coefficient km increases, the share of dust grains, *d_p_* = 1.0 µm, becomes smaller, and that of dust grains, *d_p_* = 0.7 µm, becomes larger, which indicates a systematically increasing filtration performance of the PC bed. In the last phase of the second stage of the filtration process (*k_m_* = 126–209 g/m^2^), the maximum share of dust grains *d_p_* = 0.7 µm takes on values at *U_pPC_* = 40%, which indicates the high filtration performance of the cartridge.

**Figure 26:**
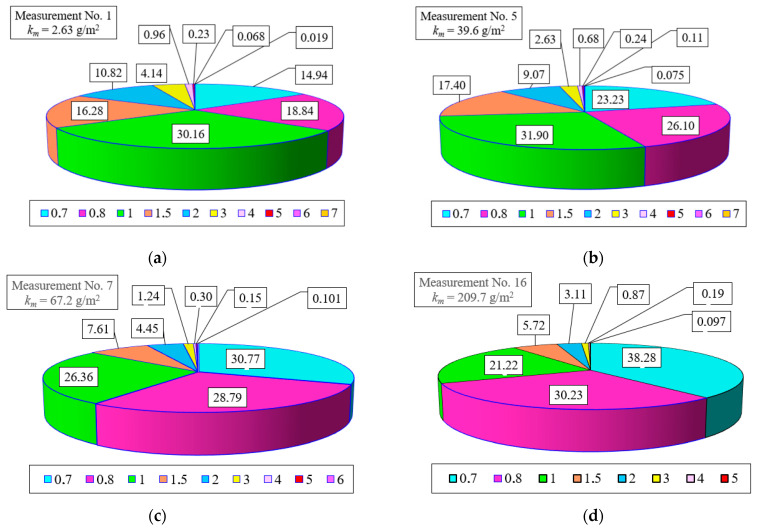
Granulometric composition of grains in the air behind the PC-2 filter after reaching successive values of dust absorption coefficients: (**a**) *k_mPC_* = 2.63 g/m^2^ (measurement No. 1), (**b**) *k_mPC_* = 39.6 g/m^2^ (measurement No. 5), (**c**) *k_mPC_* = 67.2 g/m^2^ (measurement No. 7), (**d**) *k_mPC_* = 67.2 g/m^2^ (measurement No. 16).

An example of the detailed granulometric composition of grains in the air behind the PC-2 filter for selected values of dust absorption coefficients: *k_mPC_* = 2.63 g/m^2^ (measurement No. 1), *k_mPC_* = 39.6 g/m^2^ (measurement No. 5), *k_mPC_* = 67.2 g/m^2^ (measurement No. 7), *k_mPC_* = 67.2 g/m^2^ (measurement No. 16) is shown in Figure 26a,b.

## 5. Conclusions

Experimental tests carried out with the aim of comparative evaluation of different filter materials and their shaping technologies (pleated cylindrical, PowerCore) were performed on a special test stand equipped with the needed instruments and equipment. Characteristics of separation efficiency and filtration performance, as well as pressure drop depending on the mass of dust retained on the filter—the dust absorption coefficient *k_m_*—the mass of dust retained per 1 m^2^ of filter material [g/m^2^] were performed. This allowed the characteristics to be compared with each other.

Experimental studies of the characteristics of filter materials require the use of test dust, taking a large number of measurements of the mass of dust dispensed and retained on the test and absolute filter. During the tests, certain requirements must be met and conditions maintained. It is required that the dosage of test dust is uniform during testing, which ensures a constant value of dust concentration. This is a major problem in the absence of a suitable metering device. The test dust should be properly prepared as specified by the standard. The air flow rate through the sample filter material should be constant, ensuring a constant specified filtration rate. During the test, dust is retained on the test filter and the absolute filter, which increases the flow resistance of the test system, resulting in a decrease in the air flow rate. Therefore, the air flow rate determined during the measurement must be continuously monitored and adjusted.

Filtration efficiency was determined using the mass method, in measurement cycles lasting several minutes. After each measurement cycle, the mass before and after the test—the test filter, the absolute filter and the dust dispenser—were determined using a high-precision analytical balance, and then the filtration efficiency and dust concentration in the intake air were determined.

Experimental testing of motor vehicle engine air filters is expensive, as well as labor-intensive, and requires appropriate conditions before and during measurement. Properly trained personnel are necessary. However, experimental testing is the most reliable research method.

Experimental tests of filters made of various filter materials have made it possible to recognize and compare their properties and make the following conclusions.

(1) During the experimental study, a characteristic feature of the aerosol filtration process in filtered fibrous materials was demonstrated, which is the occurrence of the initial filtration period (the time of filter operation until the separation efficiency *φ_w_* = 99.9% is reached), which is characterized by low separation efficiency, *φ_w_*, and filtration performance, *d_pmax_*, and low pressure drop, Δ*p_w_*. During this time, dust grains of large sizes were registered in the air behind the tested filters. The largest dust grain sizes (*d_pmax_* = 35 µm) were registered behind filter C (cellulose), and the smallest, *d_pmax_* = 16–18 µm, behind PowerCore G2 and P (polyester) filters, which is closely related to the filtration properties of the filter material.
(2) With the duration of the initial filtration period (I), the separation efficiency increases its value, and the grain sizes in the air behind the filter have smaller and smaller values. Once the filters reach a separation efficiency of *φ_w_* = 99.9%, the grain sizes stabilize at *d_pmax_* = 12–15 µm for filter C and *d_pmax_* = 7–9 µm for PowerCore G2, PC and P filters.
(3) The initial period of filtration is unfavorable for the operation of an internal combustion engine, as during this period there is (caused by grains above 5 µm) accelerated wear of engine components, especially the piston, piston rings and cylinder, and as a result, the life of the engine is reduced. The initial period should be as short as possible, as this has a definite effect on the accelerated wear of engine components. The most favorable in this regard is the PowerCore G2 filter, whose initial period (I) lasts until the dust load on the filter is achieved—reaching an absorption coefficient of *k_mG_* = 33.1 g/m^2^. In comparison, the initial period (I) of a cellulose filter ends when *k_mC_* = 117.3 g/m^2^ is reached.
(4) The period of basic filtration that follows the initial period (I) is characterized by a stable separation efficiency value of *φ_w_* = 99.9%, small dust grain sizes *d_pmax_* in the air downstream of the filter, and a continuously increasing pressure drop of the filter. For the same test conditions, the intensity of the increase in pressure drop is the lowest for the PowerCore G2 filter. Thus, the operating time of this filter until reaching the assumed value of the permissible resistance Δ*p_wdop_* = 3 kPa is the longest, and the mass of accumulated dust in the filter bed is the highest, taking the value *k_m_* = 355 g/m^2^. This confirms the information about the high dust absorption capacity of PowerCore G2 filters.
(5) The high dust absorbency of PowerCore G2 filters may also be due to the fact that these filters collect dust not only in the filter bed, but also inside the channels, the outlet of which is sealed. In cylindrical filters made of pleated standard filter material, depletion of the bed’s absorbency occurs and dust accumulates on its surface. This results in a sharp increase in the resistance to flow, resulting in sticking and deformation of the pleats and their instability.
(6) The results of the filtration characteristics of PowerCore filters and cylindrical pleated cartridges obtained during the empirical study are part of the information gap in the basic properties of filter materials, which are used in the construction of filter cartridges for the intake air of internal combustion engines of motor vehicles. The results obtained confirm the information on the more favorable properties of PowerCore G2 filters in terms of filtration efficiency and dust loading until permissible flow resistance is reached.

## Data Availability

Not applicable.
